# NOXA genetic amplification or pharmacologic induction primes lymphoma cells to BCL2 inhibitor-induced cell death

**DOI:** 10.1073/pnas.1806928115

**Published:** 2018-11-07

**Authors:** Yuxuan Liu, Patrizia Mondello, Tatiana Erazo, Neeta Bala Tannan, Zahra Asgari, Elisa de Stanchina, Gouri Nanjangud, Venkatraman E. Seshan, Shenqiu Wang, Hans-Guido Wendel, Anas Younes

**Affiliations:** ^a^Department of Medicine, Memorial Sloan Kettering Cancer Center, New York, NY 10065;; ^b^Antitumor Assessment Core, Memorial Sloan Kettering Cancer Center, New York, NY 10065;; ^c^Molecular Cytogenetics Core, Memorial Sloan Kettering Cancer Center, New York, NY 10065;; ^d^Department of Epidemiology and Biostatistics, Memorial Sloan Kettering Cancer Center, New York, NY 10065;; ^e^Cancer Biology and Genetics Program, Sloan Kettering Institute for Cancer Research, Memorial Sloan Kettering Cancer Center, New York, NY 10065;; ^f^Lymphoma Service, Memorial Sloan Kettering Cancer Center, New York, NY 10065

**Keywords:** BCL2, NOXA, BIM, lymphoma, apoptosis

## Abstract

Although diffuse large B cell lymphoma (DLBCL) cells widely express the BCL2 protein, they rarely respond to treatment with BCL2-selective inhibitors. Here we show that DLBCL cells harboring PMAIP1/NOXA gene amplification were highly sensitive to BCL2 small-molecule inhibitors. In these cells, BCL2 inhibition induced cell death by activating caspase 9, which was further amplified by caspase-dependent cleavage and depletion of MCL1. In DLBCL cells lacking NOXA amplification, BCL2 inhibition was associated with an increase in MCL1 protein abundance in a BIM-dependent manner, causing a decreased antilymphoma efficacy. In these cells, dual inhibition of MCL1 and BCL2 was required for enhanced killing. Pharmacologic induction of NOXA, using the histone deacetylase inhibitor panobinostat, decreased MCL1 protein abundance and increased lymphoma cell vulnerability to BCL2 inhibitors in vitro and in vivo. Our data provide a mechanistic rationale for combination strategies to disrupt lymphoma cell codependency on BCL2 and MCL1 proteins in DLBCL.

## Significance

BCL2 selective inhibitors are promising agents currently under clinical investigation for treatment of BCL2-dependent cancers. However, the clinical activity of BCL2 inhibitors in patients with diffuse large B cell lymphoma (DLBCL) has been disappointing. In this study, we identified PMAIP1/NOXA gene amplification as a marker of sensitivity to BCL2 inhibitors in DLBCL. Cells lacking NOXA amplification were less sensitive to BCL2 inhibitors due to codependency on MCL1 and BCL2 proteins. We show that pharmacologic induction of NOXA by the HDAC inhibitor panobinostat primes DLBCL to BCL2 inhibitor-induced cell death by disrupting the codependency on BCL2 and MCL1, mimicking the biologic effects of NOXA genetic amplification. Our data provide a mechanistic rationale for combining HDAC inhibitors with BCL2 inhibitors in DLBCL.

The B cell lymphoma 2 (BCL2) protein family is a key regulator of cell survival and death (1). The family has three functionally distinct subgroups: the antiapoptotic proteins (BCL2, BCL-XL, BCL-W, MCL1, and A1/BFL-1), the proapoptotic effector proteins (BAX and BAK), and the proapoptotic BH3-only proteins (BIM, PUMA, BID, BAD, BIK, BMF, NOXA, and HRK) (2, 3). Small molecules that mimic BH3 domain (BH3-mimetics) have shown a promising therapeutic value for the treatment of cancer (4, 5). BH3-mimetics bind to the antiapoptotic proteins (such as BCL2 and MCL1), liberating proapoptotic proteins and triggering apoptosis (6).

Several BH3-mimetic small-molecule inhibitors that can selectively target BCL2 were recently evaluated in patients with cancer, including venetoclax (ABT-199) and S55746 (7–9). To date, the only BCL2 inhibitor that is approved by regulatory agencies is venetoclax, which has a narrow indication focusing on patients with relapsed chronic lymphocytic lymphoma/leukemia. In more common B cell malignancies, such as diffuse large B cell lymphoma (DLBCL), BCL2 inhibitors have shown only modest clinical activity (10). Thus, identifying molecular mechanisms that may increase DLBCL vulnerability to BCL2 inhibitors is of a great clinical importance.

In this study, we investigated mechanisms of resistance to BCL2 inhibitors in DLBCL. We identified NOXA as a key player in predicting sensitivity to BCL2 inhibitors. Our study provides insights into the development of mechanism-based combinations to enhance the activity of BCL2 inhibitors in DLBCL.

## Results

### PMAIP1/NOXA Genetic Amplification Predicts Sensitivity to BCL2 Inhibitors in DLBCL Cell Lines in Vitro and in Vivo.

**Fig. 1.**
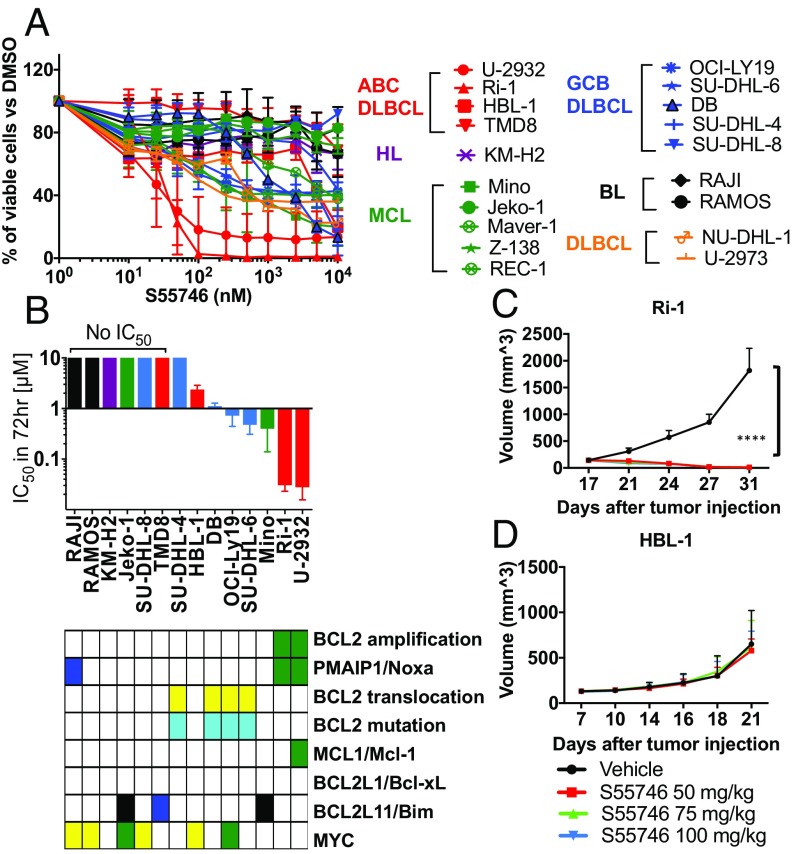
Lymphoma cells with BCL2/PMAIP1 coamplification are highly sensitive to BCL-2 inhibitor. (*A*) Dose–response curve showing the effect of pharmacological inhibition of BCL2 on lymphoma cells. Cells were incubated with S55746 from 0.01 μM to 10 μM for 72 h. Cell viability was determined by MTS assay. Cells are color-coded by tumor types (ABC DLBCL in red, GCB DLBCL in blue, MCL in green, HL in purple, and BL in black). Error bars represent SEM of triplicate experiments. (*B*) Correlation between the IC_50_ concentrations (mean ± SEM) and genetic alterations in lymphoma cell lines. The two most sensitive cell lines (U-2932 and Ri-1) harbored BCL2 and PMAIP1/NOXA gene amplifications. (*C* and *D*) In vivo activity of S55746 in two DLBCL lymphoma xenograft models. (*C*) Ri-1 (NOXA amplification) and (*D*) HBL-1 (no NOXA amplification). Mice were treated with S55746 or vehicle (intravenously) at 50, 75, or 100 mg/kg, once a day for 3 wk. Tumor volume was measured three times per week. S55746 strongly inhibits tumor growth in Ri-1 but not in HBL-1. Differences among groups were calculated with the ANOVA Dunnett’s test. ****P < 0.00001.

**Fig. 2.**
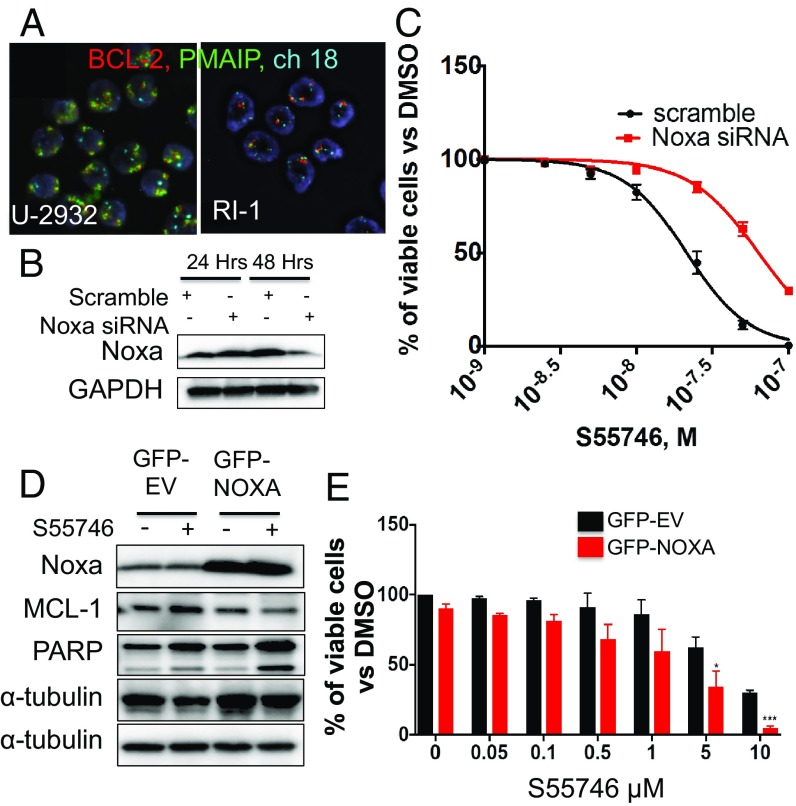
*PMAIP1/NOXA* gene amplification increases DLBCL vulnerability to BCL2 inhibitors. (*A*) Representative FISH staining in U-2932 and Ri-1 cells demonstrating an increase BCL2 (red) and PMAIP1/NOXA (green) copy numbers. Centrosome of chromosome 18 is shown in light blue. (Magnification: 63×.) (*B*) NOXA genetic silencing attenuates S55746 activity in Ri-1 DLBCL cells. Ri-1 cells were transfected with 1 μM scramble or PMAIP1/NOXA siRNA and incubated with increasing concentrations of S55746 (0.1, 0.25, and 0.5 μM). (*C*) Cell viability was assessed for cells in *B* by MTS assay after 48 h. Error bars represent SEM of triplicate experiments. Differences between groups were calculated with the Student *t* test. ***P = 0.005, ****P < 0.0001. (*D*) NOXA ectopic overexpression enhanced the efficacy of S55746 in HBL-1 DLBCL cells. (*E*) Cell viability was assessed for cells in *D* by MTS assay after 48 h. Viability data were normalized to effect of NOXA overexpression alone. Error bars represent SEM of triplicate experiments. *P < 0.05, ***P < 0.0005.

We examined the in vitro antiproliferative activity of BCL2-selective inhibitors in a wide range of lymphoma cell lines, including DLBCL, mantle cell lymphoma (MCL), Hodgkin lymphoma (HL), Burkitt lymphoma (BL), and anaplastic large cell lymphoma. The BCL2-selective small-molecule inhibitors, S55746 and venetoclax (ABT-199), demonstrated a similar activity in 26 lymphoma cell lines (*SI Appendix*, Fig. S1*A*). BCL2 protein expression was required but was not sufficient to predict sensitivity to BCL2 inhibitors (*SI Appendix*, Fig. S1*B*). Within DLBCL cell lines, Ri-1 and U-2932 were the most sensitive cells to BCL2 inhibition (Fig. 1*A* and *SI Appendix*, Fig. S1*C*). These two cell lines harbored genetic amplification of PMAIP1/NOXA and BCL2 genes (Fig. 1*B*). To validate this observation in vivo, we generated mouse xenograft models using two cell lines: Ri-1, which harbors NOXA and BCL2 gene amplifications, and HBL-1, which expresses BCL2 but very low NOXA. The presence of NOXA genetic amplification predicted sensitivity to BCL2 inhibition in the Ri-1 mouse xenograft model but not in the HBL-1 mouse xenograft model (Fig. 1 *C* and *D*). Genetic amplification of BCL2 and PMAIP1/NOXA was further confirmed in U-2932 and Ri-1 cells using FISH (Fig. 2*A*) and PCR copy number assay (*SI Appendix*, Fig. S1*D*).

NOXA is a BH3-only BCL2 family protein that promotes apoptosis by preferentially binding to MCL1 protein. NOXA gene silencing by siRNA decreased the sensitivity of Ri-1 cells to BCL2 inhibition (Fig. 2 *B* and *C*). Conversely, ectopic expression of NOXA by retroviral transduction sensitized HBL-1 cells to the BCL2 inhibitor S55746 (Fig. 2 *D* and *E*). These data confirm the association between *NOXA* gene amplification and priming DLBCL cells to BCL2 inhibitors.

**Fig. 3.**
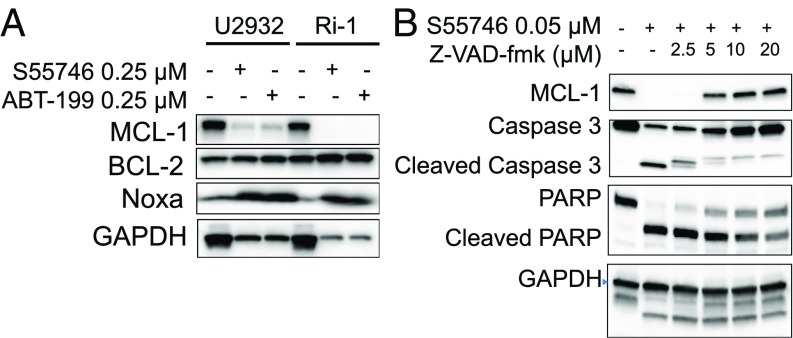
BCL2 inhibitors deplete MCL1 in DLBCL cells harboring NOXA gene amplification by a caspase-dependent mechanism. (*A*) DLBCL cells Ri-1 and U-2932 harboring NOXA genetic amplification were incubated with 0.25 μM of S55746 or venetoclax (ABT199) for 72 h. Neither drug had an effect on BCL2 protein levels, but both drugs depleted MCL1 protein. Within the time frame and drug concentrations, BCL2 inhibitors also increased NOXA protein levels in these cell lines. (*B*) S55746-induced MCL1 depletion was prevented by the pan-caspase inhibitor Z-VAD-fmk. Ri-1 cells were incubated with S55746 (0.05 μM) and increasing concentrations of ZVAD-fmk for 24 h, before protein levels were determined by Western blotting. In the absence of caspase inhibition, S55746 cleaved caspase 3 and PARP and depleted MCL1. These events were reversed by the pan-caspase inhibitor ZVAD-fmk.

In the two most sensitive cell lines (U-2932 and Ri-1), BCL2 inhibition by either S55746 or venetoclax reduced MCL1 protein abundance while increasing NOXA protein levels (Fig. 3*A*). The decrease in MCL1 protein level was due to caspase cleavage, as it was prevented by using the pan-caspase inhibitor Z-VAD-fmk (Fig. 3*B*). Thus, in these two sensitive cell lines, BCL2 inhibition induced cell death by activating the intrinsic apoptosis pathway, as demonstrated by activating caspase 9, which was further enhanced by secondary activation and cleavage of caspase 8 and MCL1 (*SI Appendix*, Fig. S2).

### BCL2 Inhibition or Genetic Silencing Is Associated with an Increase in MCL1 Protein Abundance in a BIM-Dependent Manner.

**Fig. 4.**
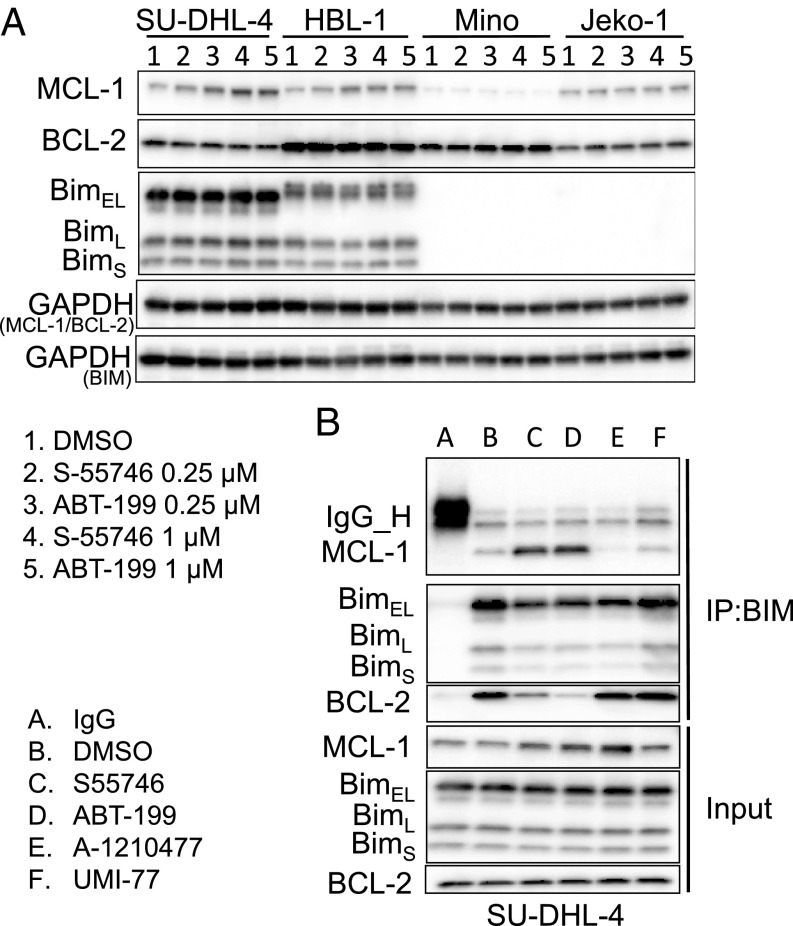
BCL2 inhibition increases MCL1 protein abundance in a BIM-dependent manner. (*A*) Western blot analysis showing the effect of BCL2 inhibitors S-55746 and ABT-199 with two doses (0.25 μM and 1 μM for 24 h) on MCL-1, BCL2, and Bim protein expression in Bim-positive cell lines (SU-DHL-4 and HBL-1) vs. Bim-negative cell lines (Mino and Jeko-1). (*B*) SU-DHL-4 cells were treated with 0.1 DMSO, 0.5 μM S-55746, 0.5 μM ABT199, 5 μM A-1210477, and 5 μM UMI-77, respectively, for 24 h. The interaction of Bim and MCL-1 was determined by immunoprecipitation (IP) and analyzed by Western blot analysis.

In DLBCL and MCL cells that lacked NOXA gene amplification, BCL2 inhibition was associated with an increase in MCL1 protein abundance (Fig. 4*A*). The increase in MCL1 protein level was observed only in cell lines that expressed BIM (Fig. 4*A* and *SI Appendix*, Fig. S3). Similarly, BCL2 genetic silencing resulted in an increase in MCL1 protein levels (*SI Appendix*, Fig. S4*A*) without changes in MCL1 transcription (*SI Appendix*, Fig. S4*B*).

BIM is a BH3-only family member that promotes apoptosis by activating downstream family members BAX and BAK. All antiapoptotic BCL2 family proteins, including MCL1, BCL2, BCLxL, and BCLw, bind to BIM, preventing it from activating BAX and BAK. BCL2 small-molecule inhibitors bind to the BH3 groove in the BCL2 protein, competing with and releasing BIM (11). Consequently, the released BIM activates BAX and BAK, inducing apoptosis. Accordingly, we investigated whether the released BIM in response to S55746 treatment may be sequestered by binding to MCL1 protein, increasing its stability and cellular abundance. Indeed, immunoprecipitation of BIM after treatment with BCL2 inhibitors was associated with a decrease in BIM association with BCL2, but with an increase in association with MCL1 (Fig. 4*B*).

Our results indicated that DLBCL cells that do not harbor NOXA gene amplification were less sensitive to BCL2 inhibitors due to codependency on MCL1 protein. To test this hypothesis, we examined whether MCL1 genetic silencing enhanced the sensitivity to BCL2 inhibitors. As shown in *SI Appendix*, Fig. S5*A*, genetic silencing of MCL1 using siRNA enhanced the antiproliferative activity of S55746 in lymphoma cell lines. Similarly, chemical inhibition of MCL1 using the small-molecule inhibitor UMI-77 (*SI Appendix*, Fig. S5*B*) or A-1210477 (*SI Appendix*, Fig. S6*A*) enhanced S55746-mediated cytotoxicity in BCL2-dependent cell lines. Furthermore, the combination of S55746 and A-1210477 was more effective in inducing caspase 3 and 7 activity compared with each drug alone (*SI Appendix*, Fig. S6*B*). In vivo inhibition of MCL1 using the small-molecule inhibitor UMI-77 also sensitized lymphoma cells to S55746 in a xenograft mouse model (*SI Appendix*, Fig. S7*A*), resulting in an increase in caspase 3 cleavage and apoptosis (*SI Appendix*, Fig. S7*B*). Taken together, our data indicate that in DLBCL that do not harbor NOXA amplification, dual inhibition of BCL2 and MCL1 will be required to induce more effective cell death. In contrast, single-agent BCL2 inhibitor is sufficient to induce cell death in lymphoma cells harboring NOXA amplification.

### The HDAC Inhibitor Panobinostat Up-Regulates NOXA and Primes DLBCL Cells to S55746-Induced Cell Death in Vitro and in Vivo.

**Fig. 5.**
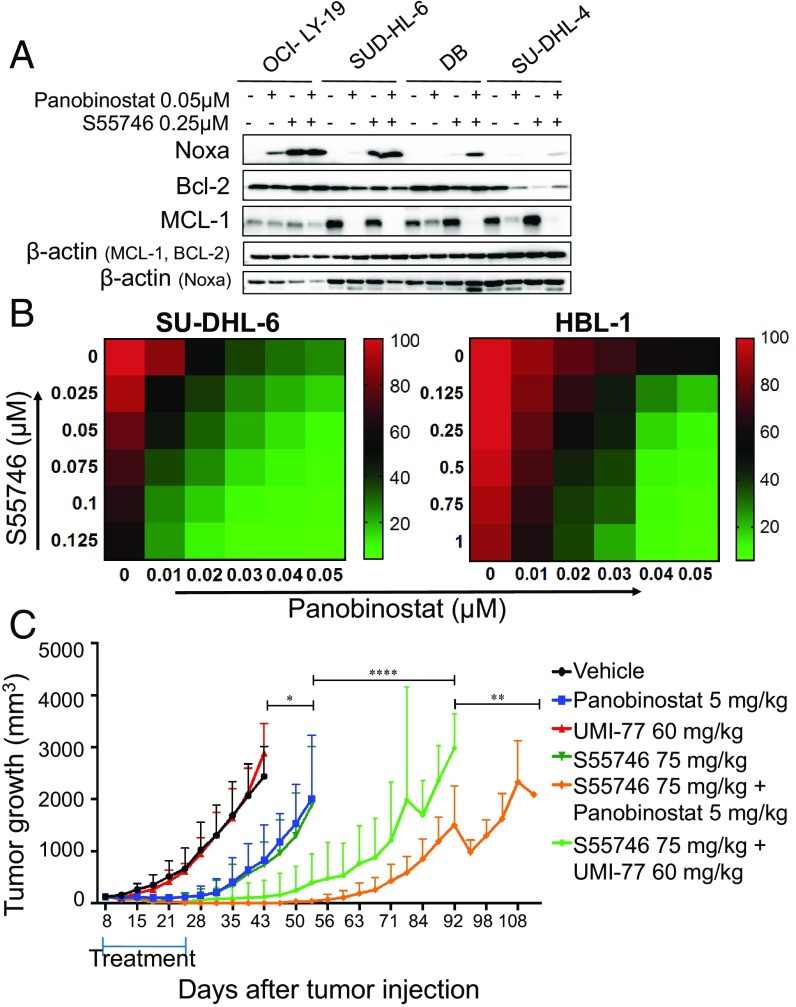
S55746 synergizes with panobinostat in vitro and in vivo in DLBCL. (*A*) Western blot showing increase in NOXA protein levels and decrease in MCL1, after treatment with the combination of panobinostat and S55746 for 24 h. (*B*) Heat maps showing the effect of the combination of S55746 and panobinostat on cell viability. Percentage of cell viability is depicted in a colorimetric scale from red (high) to green (low) normalized to DMSO (control). Values are the mean ± SD of three separate determinations. Cells were incubated with increasing concentrations of S55746 and panobinostat for 24 h and cell viability was determined by Celltiter-Glo assay. (*C*) NSG mice (*n* = 8 per treatment group) were injected with DLBCL PDX and with either vehicle, panobinostat(5 mg/kg five times weekly), UMI-77 (60 mg/kg every other day), S55746 (75 mg/kg five times weekly), or the two drugs together for 3 wk and observed until death after the end of the treatment. Differences among groups were calculated with the ANOVA with Dunnett’s test. *P = 0.04, **P = 0.003, ***P = 0.0007, ****P < 0.0001.

Previous studies demonstrated that epigenetics-modulating agents may induce apoptosis in cancer cells by modulating the expression of a variety of BCL2 family proteins (12–14). Given our observation that genetic amplification of NOXA or its forced ectopic expression by gene transfer increased sensitivity to BCL2 inhibitors, we examined whether pharmacologic up-regulation of NOXA by HDAC inhibitors may produce similar biological effects. As shown in Fig. 5*A* and *SI Appendix*, Fig. S8, the HDAC inhibitor panobinostat, alone or in combination with S55746, increased NOXA protein levels and decreased MCL1 protein levels. Furthermore, panobinostat enhanced the efficacy of the BCL2 inhibitor S55746 in several lymphoma cell lines (Fig. 5*B* and *SI Appendix*, Fig. S9).

Finally, we examined the efficacy of S55746 in combination with either the MCL1 inhibitor UMI-77 or the HDAC inhibitor panobinostat in vivo using a DLBCL PDX mouse model (Fig. 5*C* and *SI Appendix*, Fig. S10). NSG mice implanted s.c. with PDX DLBCL cells were treated i.p. daily with either vehicle, S55746 (75 mg/kg), panobinostat (5 mg/kg), or UMI-77 (60 mg/kg) three times per week. Mice were also treated with the combination of panobinostat plus S55746 or UMI-77 plus S55746. Treatment was given for three consecutive weeks, after which the animals were observed off therapy. During the 3-wk treatment period, UMI-77 showed no significant antitumor activity, while panobinostat and S55746 were equally effective (*SI Appendix*, Fig. S10*A*). Furthermore, S55746 plus UMI-77 or S55746 plus panobinostat were equally effective during the treatment period (*SI Appendix*, Fig. S10*A*). However, during the observation period after completing therapy, the combination of S55746 plus panobinostat demonstrated a longer antitumor activity (Fig. 5*C*). Tumors regrew to 2,000 mm^3^ after a mean of 85 d in animals treated with S55746 plus UMI-77 compared with 103 d in those treated with panobinostat plus S55746, which was translated into survival benefit favoring panobinostat plus S55746 combinations (*SI Appendix*, Fig. S10*B*).

## Discussion

BCL2 is a promising therapeutic target for treatment of cancer, including lymphomas. To date, venetoclax is the only small-molecule-selective BCL2 inhibitor that is approved by regulatory agencies, which narrowly focused on the treatment of chronic lymphocytic leukemia. In the more common lymphoma subtypes, such as DLBCL and follicular lymphoma, venetoclax was less active. The lack of clinical efficacy of BCL2 inhibitors in DLBCL despite the expression of the BCL2 protein target generated interest in finding vulnerabilities that can be explored in combination strategies (15). In this study, we describe a genetic alteration that enhances DLBCL vulnerability to BCL2 inhibitors and propose a mechanistic rationale for combining BCL2 inhibitors with the HDAC inhibitor panobinostat for the treatment of DLBCL.

We demonstrated that cells with coamplification of BCL2 and PMAIP/NOXA are highly sensitive to BCL2 inhibition. NOXA is a BH3-only protein that can bind and trigger proteasome-mediated MCL1 degradation (16). In our study, we showed that genetic silencing of NOXA decreased sensitivity to S55746; in contrast, overexpression of NOXA by gene transfer sensitized DLBCL cells to S55746. In recent clinical trials, venetoclax showed a modest clinical activity in patients with relapsed DLBCL (17). Given the need for predictive biomarkers for selection of patients, it would be important to examine tissue specimens from patients enrolled in these clinical trials to determine whether responding tumors present BCL2/NOXA amplification. However, this is unlikely given the rare incidence of NOXA mutations in primary DLBCL tumors.

The antiapoptotic proteins BCL2 and MCL1 can sequester BIM, a proapoptotic BH3-only family member. Liberating BIM is important for directly activating the proapoptotic proteins BAX and BAK and inducing apoptosis. S55746, a BH3-mimetic small molecule, released BIM from binding to BCL2. However, the released BIM is rapidly sequestered by MCL1, preventing it from activating BAX/BAK. Furthermore, BIM stabilizes MCL1, inhibiting its degradation and causing an increase in MCL1 protein abundance.

**Fig. 6.**
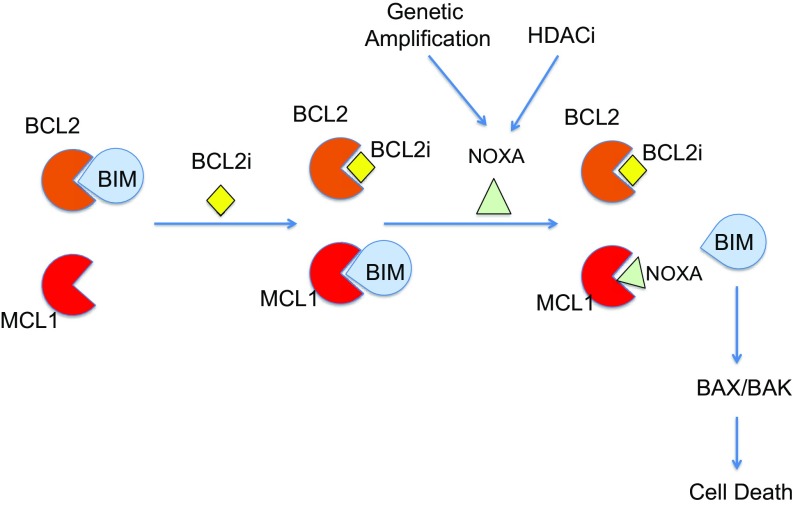
A model for dual inhibition of BCL2 and MCL1 to release BIM and induce apoptosis. BCL2 protein function can be inhibited by either venetoclax or S55746, releasing BIM from binding to BCL2. BIM is subsequently sequestered by MCL1. BIM is released from MCL1 by NOXA, through genetic amplification or pharmacologic induction by the HDAC inhibitor panobinostat. The free BIM can directly activate BAX/BAK and induce cell death.

We found that BIM is frequently expressed in our DLBCL cell lines. In fact, based on the Cancer Cell Line Encyclopedia, DLBCL lines express the highest mRNA expression level of BCL2L11/BIM compared with other cancer cell lines (*SI Appendix*, Fig. S11). Given that both BCL2 and MCL1 can bind and sequester BIM (Fig. 4*B*) and MCL1 knockdown enhances S55746 antiproliferative activity, these data suggest that dual inhibition of both BCL2 and MCL1 may release an adequate amount of BIM to activate BAX/BAK and induce apoptosis (Fig. 6).

We showed that cell lines that harbored genetic amplification of *NOXA* were very sensitive to S55746-induced cell death. Pharmacologic induction of NOXA using the HDAC inhibitor panobinostat also enhanced lymphoma cell sensitivity to S55746. An alternative strategy for dual targeting of BCL2 and MCL1 was recently reported, demonstrating a synergistic induction of apoptosis by combining venetoclax with the cyclin-dependent kinase inhibitors dinaciclib or flavopiridol (18, 19). Other groups have shown that the balance between NOXA and MCL1 regulates sensitivity to BH3-mimetics and that drugs such as dasatinib, fludarabine, bortezomib, and etoposide can similarly modulate NOXA and MCL1 levels (20–22). With the recent development of clinical-grade selective MCL1 inhibitors, it would be important to determine whether the systemic combination of BCL2 and MCL1 inhibitors is safe in the clinical setting (23).

Our study demonstrated that the expression of BCL2 was required but was not sufficient to predict sensitivity to BCL2 inhibitors. However, it is difficult to compare the level of drug sensitivity across several published studies, mainly due to differences in cell line characteristics, passages, and experimental methods. In our study, all cell lines were genetically authenticated, and all experiments were performed in cell lines with a low number of passages. Furthermore, drug resistance was confirmed using two independent methods (Fig. 1*A* and *SI Appendix*, Fig. S1).

In conclusion, our findings provide insights on the mechanism of action of S55746 and provide a mechanistic rational for combining panobinostat with BCL2 inhibitors for the treatment of lymphoma.

## Methods

### Cell Lines.

The human DLBCL-derived cell lines SU-DHL-4, SU-DHL-6, OCI-LY19, U-2932, and Ri-1 were obtained from the German Collection of Microorganism and Cell Cultures, Department of Human and Animal Cell Cultures; DB, SU-DHL-8, and the BL cell lines RAJI, Ramos, and Daudi were obtained from ATCC. The DLBCL-derived cell lines (HBL-1 and TMD8) were provided by R. E. Davis, MD Anderson Cancer Center, Houston. Cell lines were cultured in RPMI medium 1640 supplemented with 10 or 20% heat-inactivated FBS (Gibco BRL), 1% l-glutamine, and penicillin–streptomycin in a humid environment of 5% CO_2_ at 37 °C.

### Drugs.

S55746 was obtained from Servier; A1210477 from Active Biochem; and ABT-199, UMI-77, and Z-VAD-fmk from Selleck Chemicals.

### High-Throughput Screening Experiments.

For the single-agent studies, 1 μL of compounds was preplated in a 12-point doubling dilution series with 10 μM compound concentration as the upper limit and transferred from an intermediate 384-well polypropylene microtiter plate (Thermo Scientific) to a 1,536-well microtiter assay plate (Corning) using the custom-designed 384 head on a PP-384-M Personal Pipettor (Apricot Designs).

### In Vitro Proliferation Assay.

Cells were seeded in 96-well plates at 25,000 cells per 100 μL per well or in 24-well plates at 250,000 cells per 1 mL per well with either vehicle (DMSO 0.1%) or increasing concentrations of drugs for 24, 48, and 72 h. Cell viability was assessed by adding MTS reagent or CellTiter-Glo reagent (Promega) to the culture medium at 1:5 ratios, according to the manufacturer’s instructions. Procedures to determine the effects of certain conditions on cell proliferation and apoptosis were performed in three independent experiments. The two-tailed Student *t* test and Wilcoxon rank test were used to estimate the statistical significance of differences between results from the three experiments. Significance was set at *P* < 0.05. The PRISM software was used for the statistical analyses.

### Targeted Sequencing.

To characterize lymphoma cell lines for somatic base mutations and copy number alterations in all key cancer-associated genes, we performed a custom, targeted deep-sequencing assay on cell line samples. Our assay (IMPACT) involves massively parallel sequencing, coupled with solution-phase exon capture (24, 25). Exon capture was performed on barcoded pools of sequence libraries by hybridization (Nimblegen SeqCap Target Enrichment) using custom oligonucleotides to capture all exons and select introns of 585 cancer genes, including all genes significantly mutated in hematologic malignancies. Barcoded pools were subsequently sequenced on an Illumina HiSeq 2500 to 500–1,000× coverage per sample to maximize sensitivity for detecting low-abundance alterations. Through many iterations of the design of the capture probe set, we have maximized the coverage uniformity across all exons in our panel, thus reducing the number of poorly covered exons. As a result, for a sample sequenced by HEMEPACT to 900× coverage, >98% of target exons are covered at >100×.

A pool of disease-free, frozen normal samples from 10 individuals was used as a control for processing from library preparation all the way through to sequencing. Besides helping to identify potential sequencing artifacts it also helps to filter out a number of germline variants when run in the pipeline alongside the samples.

### FISH.

Cells (2.5 × 10^5^, 250 μL) were transferred to the chambered-slide and spin down at 1,300 rpm for 5 min at 4 °C. Cells were fixed using 4% PFA for 30 min, washed by PBS, and air-dried. FISH analysis was performed using a three-color mix probe for Cen18 (aqua), BCL2 (red), and PMAIP1 (green); all three probes were purchased from Empire Genomics. Hybridization, washing, and fluorescence detection were performed according to standard procedures. Slides were scanned using a Zeiss Axioplan 2i epifluorescence microscope equipped with a megapixel CCD camera (CV-M4+CL; JAI) controlled by Isis 5.5.9 imaging software (MetaSystems Group Inc.). Signal counts performed on the captured images and a minimum of 50 nonoverlapping nuclei analyzed. Amplification was defined as BCL2 or PMAIP1:Cen18 (control) ratio of 2.2 or >10 copies of BCL2 or PMAIP1 independent of control locus or at least one small cluster of BCL2 or PMAIP1 (4 copies; resulting from tandem repeat/duplication). Cells with 3∼5 copies and 6∼10 copies of BCL2 or PMAIP1 and Cen18 were considered to be polysomic and high-polysomic, respectively.

### Quantitative RT-PCR.

Total RNA was extracted with Qiagen RNeasy mini kit. A total of 1 μg of RNA was converted to cDNA using iScript cDNA synthesis kit (Bio-Rad). Real-time PCR was performed using the model CFX96 (Bio-Rad). Primers for MCL1 and GAPDH were purchased from Bio-Rad: GAPDH (qHsaCED0038674) and MCL1 (qHsaCED0036603). SsoAdvanced Universal SYBR Green Supermix (172-5270; Bio-Rad) was used for qPCR.

### Copy Number PCR Assay.

Genomic DNA from cell lines was extracted with Qiagen DNeasy blood and tissue kit by following the instructions and was measured by NanoDrop 3300. Copy number assays (Applied Biosystems by Life Technologies) were performed by following the manufacturer’s instructions. Briefly, 20 ng of 4 μL DNA (5 ng/μL) was mixed with TaqMan Genotyping Master Mix (4371353), BCL2 (Hs01500302_cn), or PMAIP1 (Hs01670847) TaqTaqMan Copy Number Assay (20×), human TaqMan Copy Number Reference Assay RNase P (4401631) in 96-well plate. Human Genomic DNA (G1471; Promega) was used as control. CFX96 (Bio-Rad) was used for PCR. CopyCaller Software (PN 4412907; Applied Biosystems) was used to analyze the copy number experiments.

### Immunoprecipitation and Western Blotting.

Preparation of cellular protein lysates was performed by using the Cell Signaling lysis buffer (9803) according to the manufacturer’s extraction protocol. Protein quantitation was done using the Direct Detect system (Millipore). A total of 30 μg of protein was denatured in Laemmli buffer at 95 °C for 5 min and western immunoblotting was performed using the Bio-Rad system (TGX 4–15% gels). Transfer was performed using the Trans Blot turbo system (Bio-Rad) onto PVDF membranes. The immunoblotting was performed with the primary antibodies mentioned below. For immunoprecipitation, protein lysates were incubated with primary antibody overnight at 4 °C, then an equal amount of protein G agarose beads (Abcam) was added to all samples, followed by 3 h of incubation at 4 °C. The beads were washed three times with lysis buffer, eluted with Laemli buffer, and Western blotting was performed as mentioned above.

For detecting MCL1 and Bim binding, Bim was immunoprecipitated with Bim antibody (BD Biosciences) and probed with Bim antibody (Cell Signaling Technology).

Primary antibodies used were for BCL2, BCL-xL, MCL1, BIM, Bak, PUMA, caspase 3, 7, and 9, PARP, BID, GAPDH (Cell Signaling Technologies), NOXA, c-myc (Abcam), and β-actin (Sigma). Secondary anti-rabbit and anti-mouse HRP-conjugated antibodies were purchased from Bio-Rad (170-6515 and 170-6516). Images were acquired by using the Bio-Rad Imaging Chemidoc MP system. ImageJ software was used to perform densitometry analyses of Western blots. Results for each band were normalized to the beta-actin/GAPDH levels in the same blot.

### siRNA Experiments.

siRNA transfections were performed by using the Amaxa 4D-Nucleofector Unit (Lonza). Briefly, 3 × 10^6^ cells per condition were transfected with 1–2 μM siRNA or scramble and resuspended in 10% FBS RPMI with no antibiotics. siRNAs were purchased from Life Technologies: MCL1 (s8583), BCL2L11 (s195011), and Negative Control 1 (4390843) and from Dharmacon, GE Lifesciences: ON-TARGETplus Human BCL2 siRNA SMARTpool (L-003307-00-0005) and ON-TARGETplus Nontargeting Pool (D-001810-10-20). Cells were transfected using the EN-150 (Ri-1 and HBL-1) and EN-138 (TMD-8) electroporation program, respectively, using Solution F. Optimization was done using the Amaxa Cell Line Optimization 4D-Nucleofector X kit (V4XC-9064; Lonza). Transfection was performed using the 4D-Nucleofector X kit L (V4XC-2024; Lonza).

### NOXA Overexpression.

#### Retrovirus generation.

To produce retrovirus overexpressing human NOXA, 4 × 10^6^ Phoenix cells were culture in a 10-cm dish for 24 h then transfected with MSCV-IRES-GFP empty vector or NOXA. Twenty-four hours after transfection the virus-containing culture supernatant was harvested daily and kept in Retro-X concentrator (631455; Clontech). Supernatants were centrifuged at 1,500 × *g* for 45 min at 4 °C. Pellets were resuspended in complete media, aliquoted, and kept at −80 °C.

#### Transduction.

HBL-1 cells (1.0 × 10^6^) were transduced with 0.5 mL retrovirus encoding GFP-NOXA or GFP-EV (empty vector) with polybrene (4 μg/mL). Forty-eight-hoursmedia containing virus was replaced and 5 d posttransduction GFP-positive cells were sorted and expanded.

### Immunofluorescence Assay.

SU-DH-L-6 cells were stained using a Discovery XT processor (Ventana Medical Systems) in the Memorial Sloan Kettering Cancer Center Molecular Cytology Core Facility. The slides were deparaffinized with EZPrep buffer (Ventana Medical Systems), antigen retrieval was performed with CC1 buffer (Ventana Medical Systems), and sections were blocked for 30 min with Background Buster solution (Innovex) followed by Avidin/biotin blocking for 8 min. Anti-Cleaved Caspase 3 (9661, 0.1 μg/mL; Cell Signaling) antibodies were applied and sections were incubated for 5 h, followed by 60 min incubation with biotinylated goat anti-rabbit IgG (PK6101; Vector Laboratories) at 1:200 dilution. The detection was performed with a DAB detection kit (Ventana Medical Systems) according to the manufacturer’s instructions. Slides were counterstained with hematoxylin and coverslipped with Permount (Fisher Scientific). Tissue sections were digitally scanned using Pannoramic Flash (3DHistech) with a 20×/0.8 N.A. objective. Slides were then reviewed using the Pannoramic Viewer (3DHistech) software and then images were taken and exported into TIFF files at both 20× and 51× (1:1) magnification.

#### Xenograft studies.

NSG mice (The Jackson Laboratory) were used for in vivo studies. Six-week-old female mice were injected s.c. with either 10 million Ri-1 cells or 10 million PDX DLBCL cells together with matrigel. Once tumors reached an average volume of 100 mm^3^, mice were randomized to receive either vehicle control, Bcl2 inhibitor (S55746), panobinostat, or Mcl-1 inhibitor (UMI-77). Bcl2 inhibitor was dosed at either 50 mg/kg or 75 mg/kg, i.p. daily, five times weekly, panobinostat was dosed at 5 mg/kg i.p. daily, five times weekly for up to 3 wk, and Mcl-1 inhibitor was dosed at 75 mg/kg, three times per week for up to 3 wk. Mice were observed daily throughout the treatment period for signs of morbidity/mortality. Tumors were measured twice weekly using calipers, and volume was calculated using the formula: length × width^2^ × 0.52. Body weight was also assessed twice weekly. Upon treatment end, mice were monitored twice weekly for tumor regrowth and clinical signs.

#### Study approval.

The present xenograft studies were reviewed and approved by the Memorial Sloan-Kettering Cancer Center Institutional Animal Care and Use Commitee.

#### Statistical analysis.

Statistical significance was determined either by two-tailed Student *t* test or the ANOVA test. *P* values <0.05 were considered significant (*P < 0.05; **P < 0.01; ***P < 0.001; ****P < 0.0001). Survival was estimated with the Kaplan–Meier survival curve method and differences in survival were calculated by long-rank test (Graph Pad Prism 6.0).

### HTS Statistical Analysis.

The drugs were tested for activity both as single agents (S55746 and venetoclax/ABT-199) on multiple cell lines through the high-throughput screening core facility (HTSCF). The residual cell viability posttreatment with specific drug combinations was assessed in an alamarBlue assay and quantified as fluorescence signal intensity measured using the LEADseeker Multimodality Imaging System (GE Healthcare). The data were converted into percent inhibitions conferred by each combination relative to both the high (1% DMSO vol/vol) and the low (1 μM killer mix) control averages (*μ*). Of note, “killer mix” consists of a HTSCF proprietary mixture of cytotoxic compounds. The percent inhibitions were defined as:

$$\%\mathrm{inhi}b_{i}=\frac{\left(\mu_{high\;control}-value_{i}\right)}{\left(\mu_{high\;control}-\mu_{low\;control}\right)}\times 100.$$

Since the percent inhibition is derived from data measured with error some of the computed numbers can fall outside the [0, 100]% interval. We replaced such values by the appropriate boundary value. We used the average percent inhibition of the replicates at a dose level (single agent or combination) as the activity at that dose level.

To evaluate whether a drug combination shows synergy we compared the observed activity at the combination at that level to the expected activity under the Bliss independence model. By treating percent inhibition as a probability and using the product rule for the probability of independent events, the expected activity can be written as

$$P\left(Inh|A,B\right)=1-P\left(\bar{Inh}|A,B\right)=1-P\left(\bar{Inh}|A\right)\times P\left(\bar{Inh}|B\right)=1-\left[1-P\left(Inh|A\right)\right]\times \left[1-P\left(Inh|B\right)\right],$$

where *A* and *B* are the two drugs and Inh and $\bar{Inh}$ denote inhibited and not inhibited, respectively (26, 27). The observed can be compared with the expected activity using a simple difference where values around 0 represent additive relationship; large positive values represent synergy and large negative values antagonism. However, the same magnitude of the difference represents different relative change depending on the expected activity. Thus, we also use a log-odds measure given as $\log\left[P_{o}\left(1-P_{e}\right)/P_{e}\left(1-P_{o}\right)\right]$, where $P_{o}$ and $P_{e}$ are observed and expected activities. For each drug combination we generated a heat map for the observed inhibition, the difference between observed and expected inhibition, and the log-odds of the observed to expected inhibition. The observed and expected inhibition values were rescaled before calculating the log-odds by the function (0.8 × observed/expected + 0.1); this was done to adjust for instances in which the observed or expected inhibition was 0 or 1. These numbers are binned into intervals suitable for each scale and color-coded for simple visualization of the combined activity. For comparing drug combinations we combined the log-odds data across all cell lines and used the medians to rank the combinations. The data are shown as boxplots. All analyses were done using the R programming language.

## Supplementary Material

Supplementary File
