# Supplementary material for: NOXA genetic amplification or pharmacologic induction primes lymphoma cells to BCL2 inhibitor-induced cell death
Source: Proc Natl Acad Sci U S A. 2018 Nov 7;115(47):12034–9. doi: 10.1073/pnas.1806928115 (PMC6255185; doi:10.1073/pnas.1806928115)
Supplement: Supplementary File [file pnas.1806928115.sapp.pdf]

Supplementary Figure 1

A

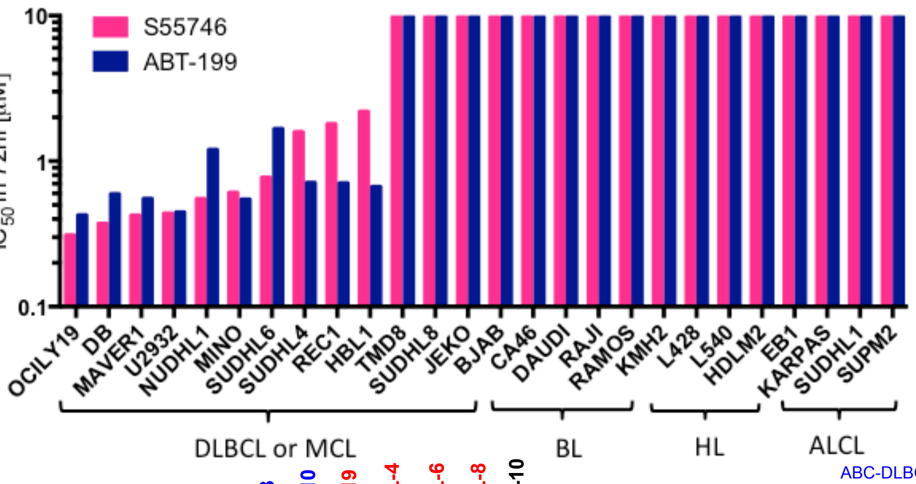

B

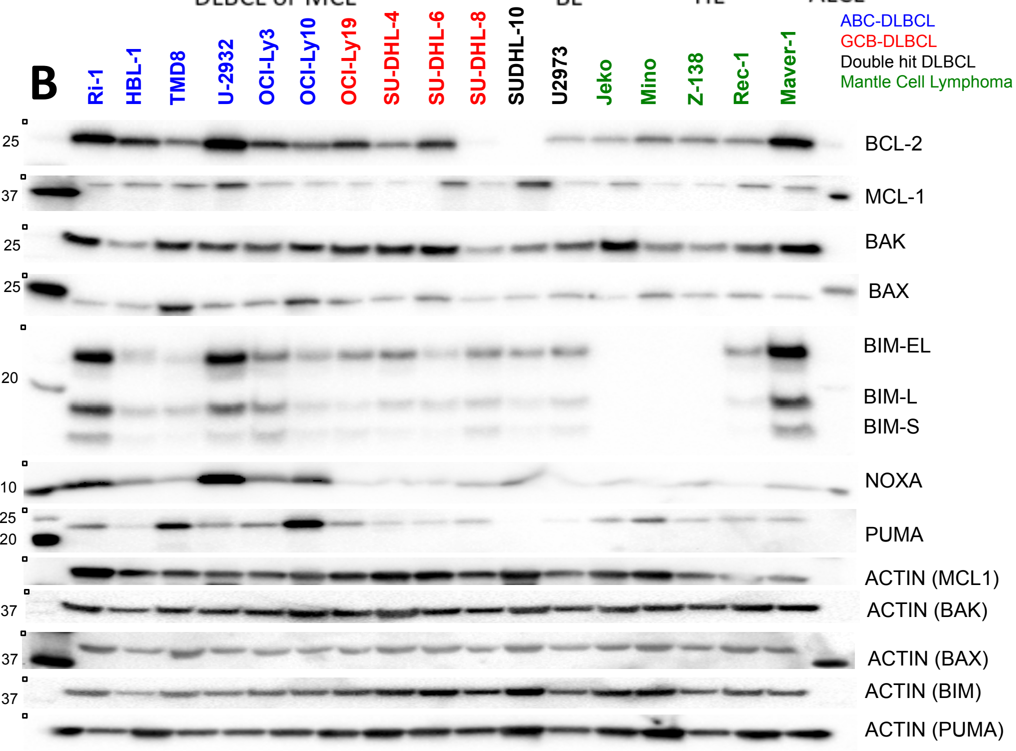

C

Cell Line	IC50 72 Hrs (μM)
U-2932	0.02
Ri-1	0.03
HBL-1	2.37
TMD8	>10
KM-H2	>10
Mino	0.23
Jeko-1	>10
Maver-1	0.45
Z-138	>10
REC-1	3.48
OCI-Ly19	0.57
SUDHL-6	0.40
DB	1.15
SUDHL-4	>10
SUDHL-8	>10
RAJI	>10
Ramos	>10
U-2973	0.22
NU-DHL-1	0.27

D

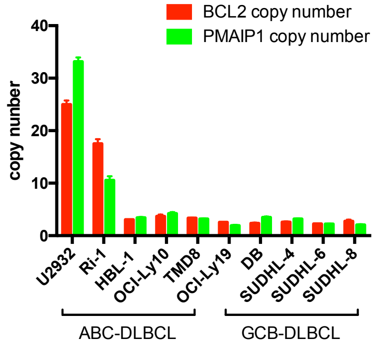

Figure S1. BCL-2 expression is required but not sufficient to predict sensitivity to BCL2 inhibitors
(A) Bar graph showing 3-day IC_{50} values in B-cell lymphoma cell lines (n=26), treated with either S-55746 or ABT-199/venetoclax in a logarithmic scale. Results are from a high-throughput screening assay of single agent dose response. DLBCL, diffuse large B cell lymphoma; MCL, mantle cell lymphoma; BL, Burkitt lymphoma; HL, Hodgkin lymphoma; ALCL, anaplastic large cell lymphoma.
(B) Western blot analysis showing baseline expression levels of BCL2 family proteins (Bcl-2, Bcl-xL, Bax, Bak, Mcl-1, Noxa, Puma and Bim). Cells are color-coded by tumor-types.
(C) Table summarizing the S55746 IC50 concentrations at 72 hours for the 19 cell lines shown in Fig 1A.
(D) BCL2 (red) and PMAIP1/NOXA (green) copy numbers in 10 DLBCL cell lines, as determined by a copy number PCR assay

Supplementary Figure 2

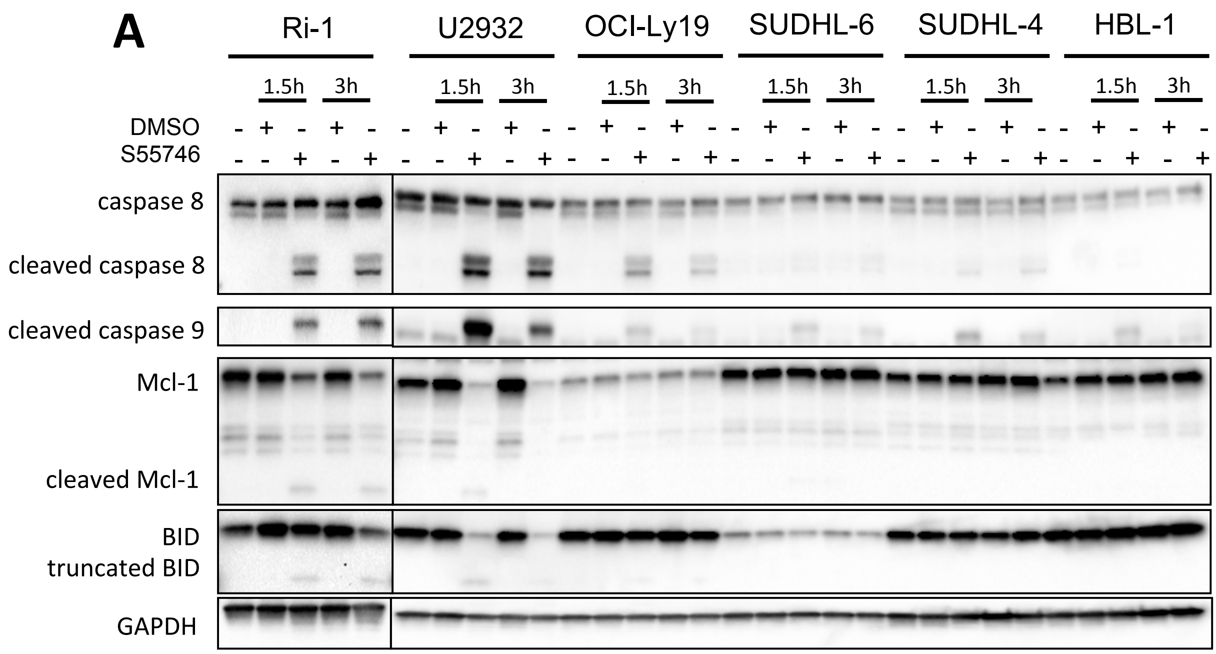

Figure S2. In vitro efficacy of the BCL2 inhibitor S55746 in cell lines harboring NOXA/BCL2 gene amplification.

(A) Western blot showing that sensitivity to S55476 in Ri-1 and U2932 cells is associated with, activation of caspase 8, 9 and depletion of MCL1 protein

Supplementary Figure 3

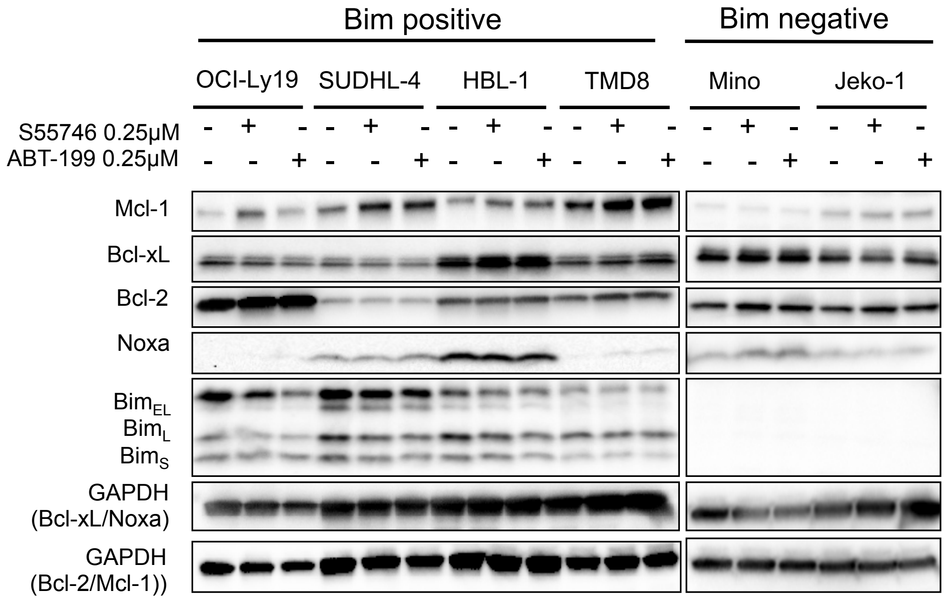

Figure S3. BCL2 inhibitors induce an increase in MCL-1 levels in Bim positive cell lines

Western blot analysis showing the effect of BCL2 inhibitors S-55746 and ABT-199/venetoclax (0.25 μM for 24 hours) on Bcl2 family proteins I Bim positive and Bim negative cell lines.

Supplementary Figure 4

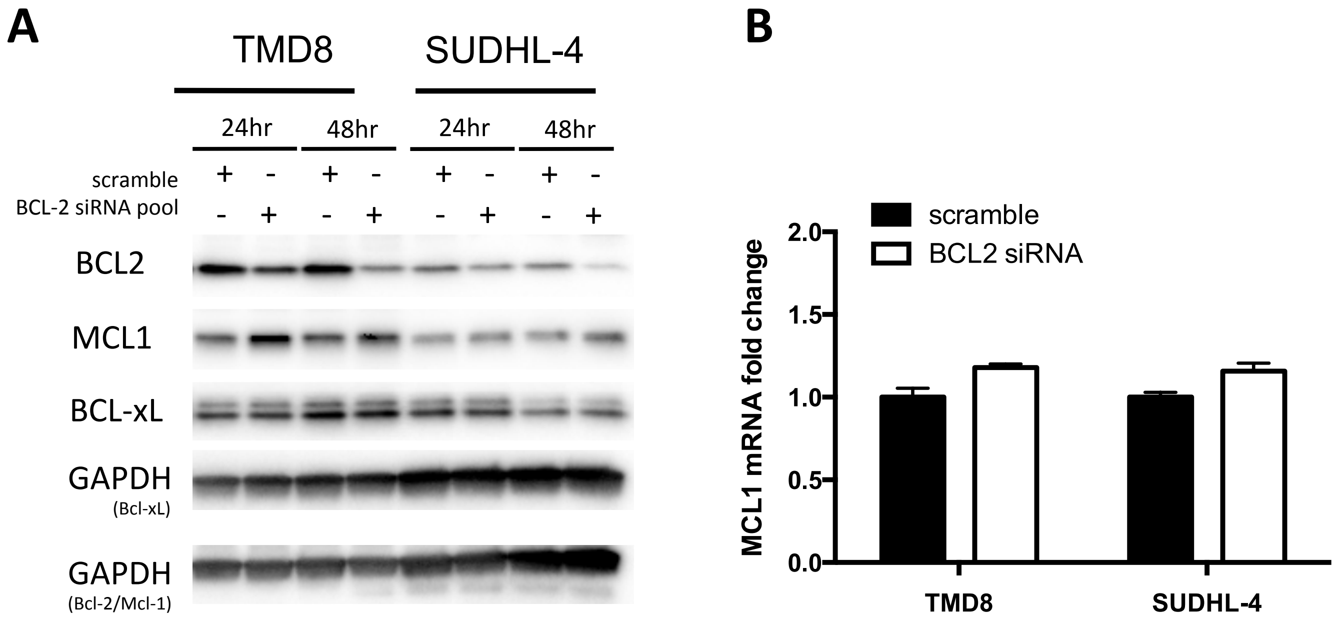

Figure S4. BCL2 gene silencing is associated with an increase in MCL1 protein abundance in DLBCL

A. Western blot analysis showing the effect of BCL2 depletion by RNA interference on MCL1 and BCL2, and BCL-xL in 2 representative lines (TMD8 and SUDHL-4). Cells were transfected either with scramble siRNA or BCL2 siRNA for the indicated time.

B. BCL2 gene silencing had no significant effect on the mRNA levels of MCL1 in TMD8 and SUDHL-4

Supplementary Figure 5

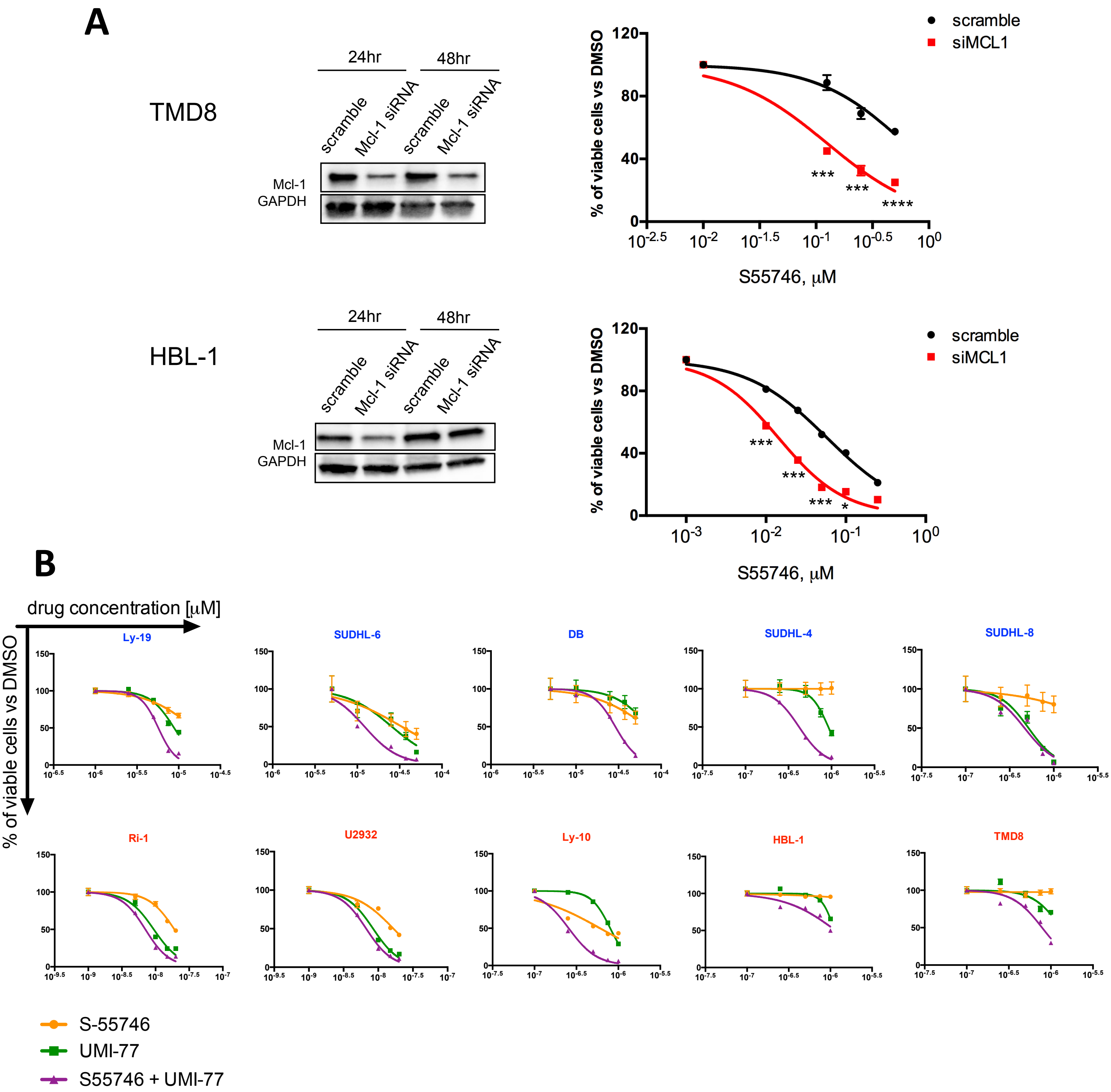

Figure S5. MCL1 gene silencing or functional inhibition enhances sensitivity to BCL2 inhibitors

A. Cells were transfected either with scramble siRNA or MCL1 siRNA for the indicated time. Western blot confirming MCL1 depletion by RNA interference in 2 representative lines (TMD8 and HBL-1). MTS assay of cells transfected with scramble or MCL1 siRNA treated with different doses of S-55746 for 24h.

B. MTS assay confirming enhanced antiproliferative effect of BCL2 inhibitor S-55746 in combination with MCL-1 inhibitor UMI-77 in DLBCL cell lines. Cells were treated with increasing concentrations of S-55746 in the presence or absence of increasing concentration of UMI-77 for 48 hrs. All data points represent the means of triplicate experiments, with error bars indicating the S.E.M.

Supplementary Figure 6

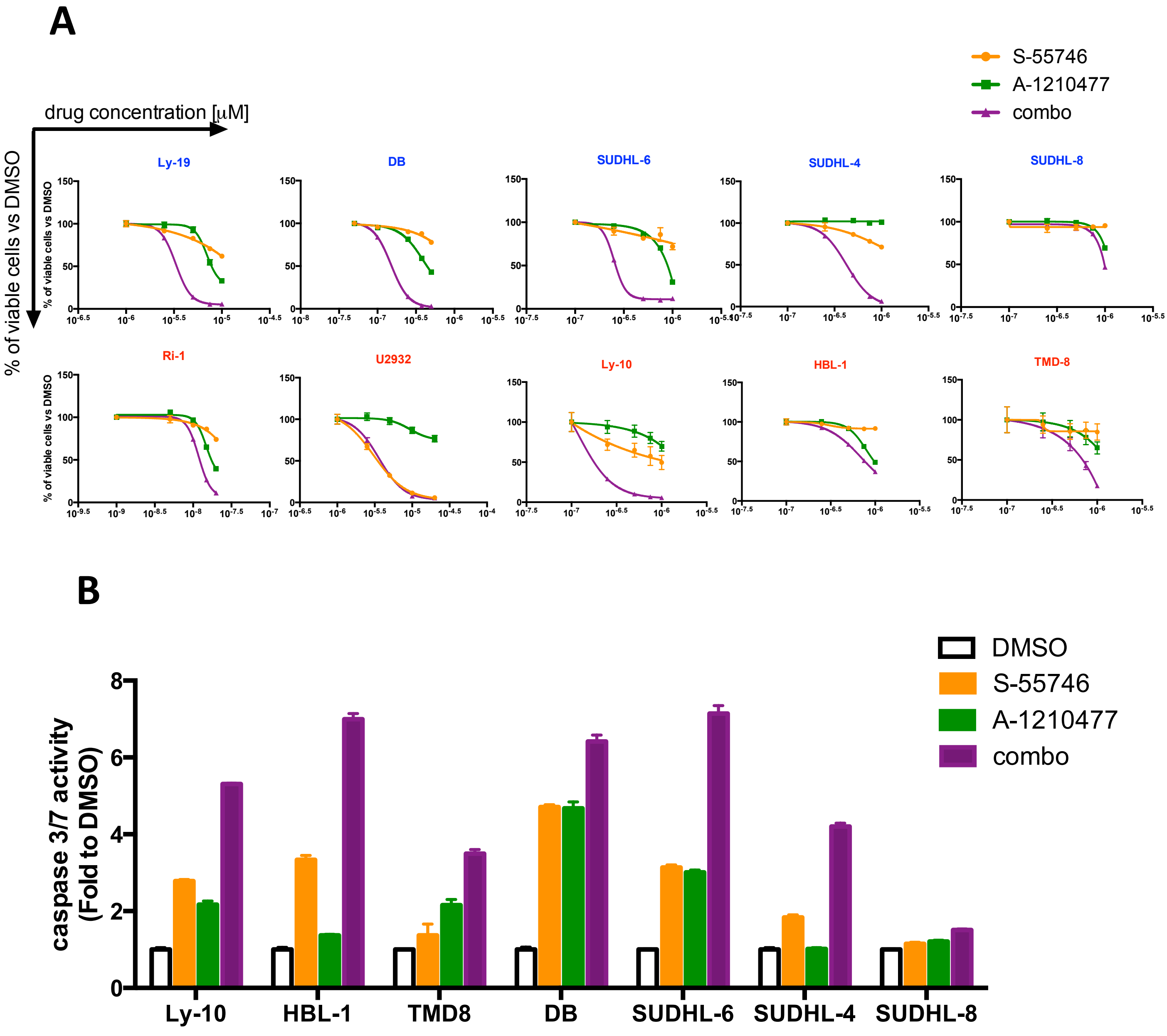

Figure S6. MCL-1 small molecule inhibitor A1210477 enhances S55476 activity and induces caspase 3/7 activation.

A. MTS assay confirming enhanced antiproliferative effect of BCL2 inhibitor S-55746 in combination with MCL-1 inhibitor A-1210477 in DLBCL cell lines. Cells were treated with increasing concentrations of S-55746 in the presence or absence of increasing concentration of A-1210477 for 48 hrs. All data points represent the means of triplicate experiments, with error bars indicating the S.E.M.

B. Cells were treated with S-55746 (yellow) in the presence or absence of A-1210477 (green) for 18 hours before assessing caspase 3/7 activities by immunohistochemistry

Supplementary Figure 7

A

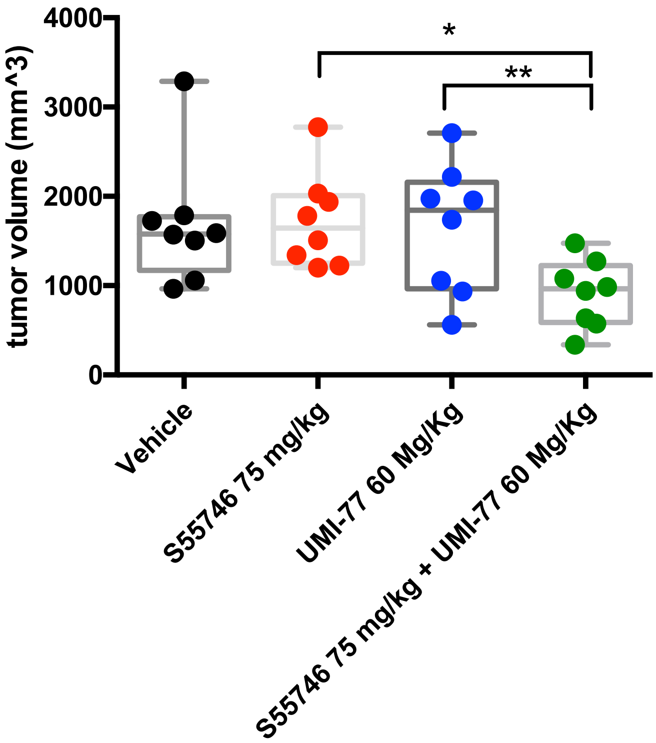

B

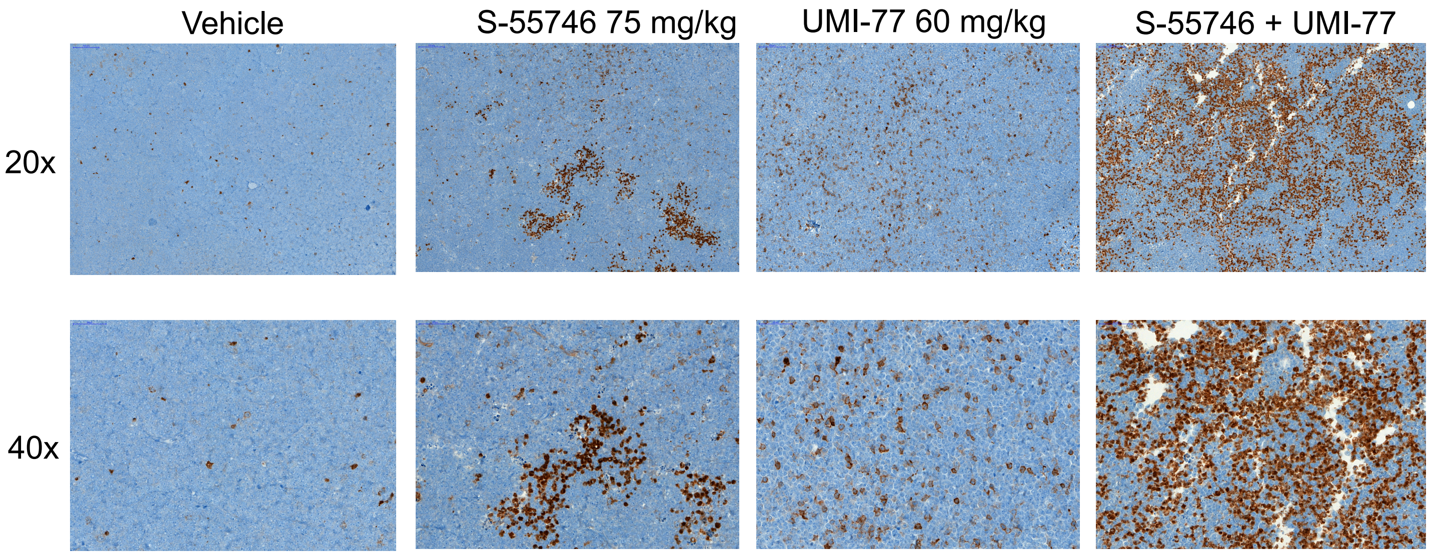

Figure S7. The MCL1 small molecule inhibitor UMI-77 enhances the antiproliferative activity of the BCL2 inhibitor S-55746 in vivo

A. NSG mice (n = 8 per treatment group) bearing SUDHL-6 tumors were treated intravenously with either vehicle, S-55746 (75 mg/kg once a day), UMI-77 (60 mg/kg every other day) or the two drugs together for 3 weeks. Tumor volumes were measured 3 times per week. Differences between groups were calculated with the ANOVA with Dunnett's test. *$P< 0.05$, **$P < 0.005$.

B. Immunohistochemistry staining showing cleaved caspase 3 protein expression in SUDHL-6 xenografts after administration of either vehicle, S-55746 at 75 mg/kg, UMI-77 at 60 mg k or the combination of the two drugs for 3 weeks.

Supplementary Figure 8

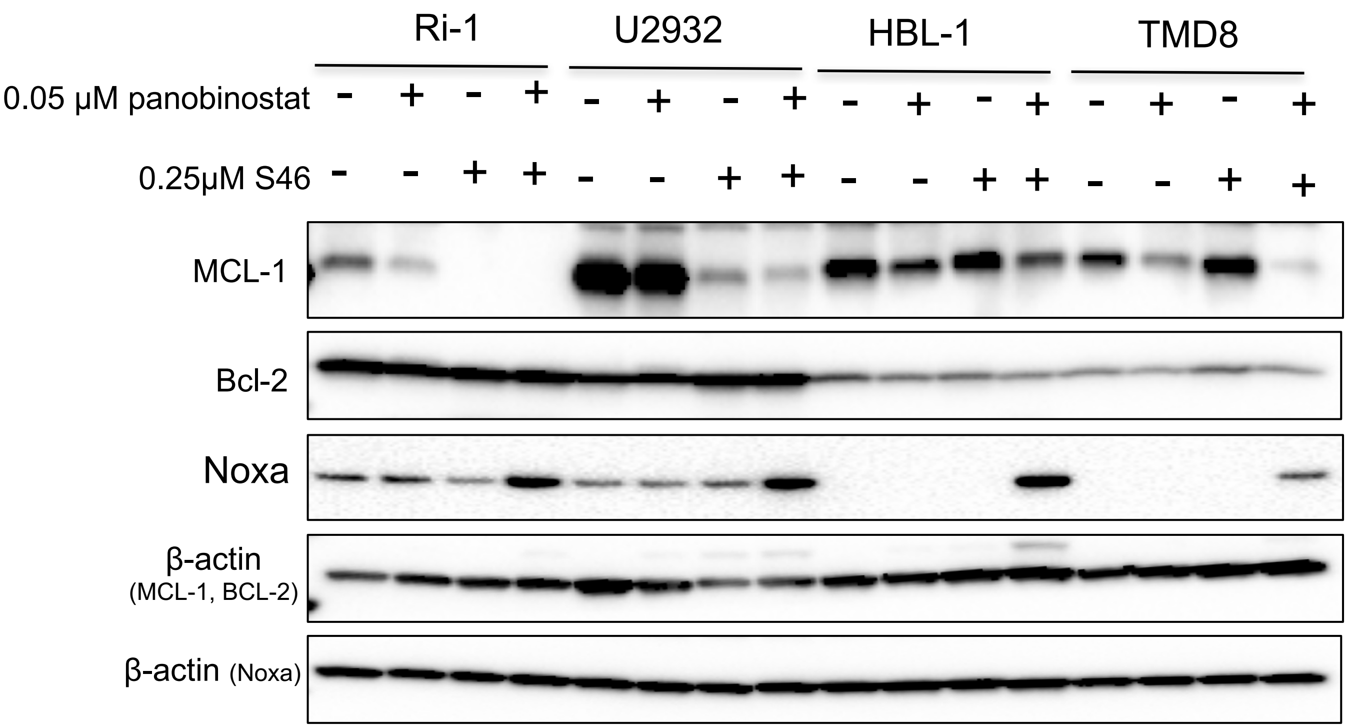

Figure S8. Effect of S-55746 and panobinostat on MCL1 and NOXA protein levels in DLBCL. Western blot showing enhanced decrease in MCL1 protein level and increase in NOXA protein level after treatment with the combination of panobinostat and S-55746 for 24 hours. The drug had no effect on BCL2 levels.

Supplementary Figure 9

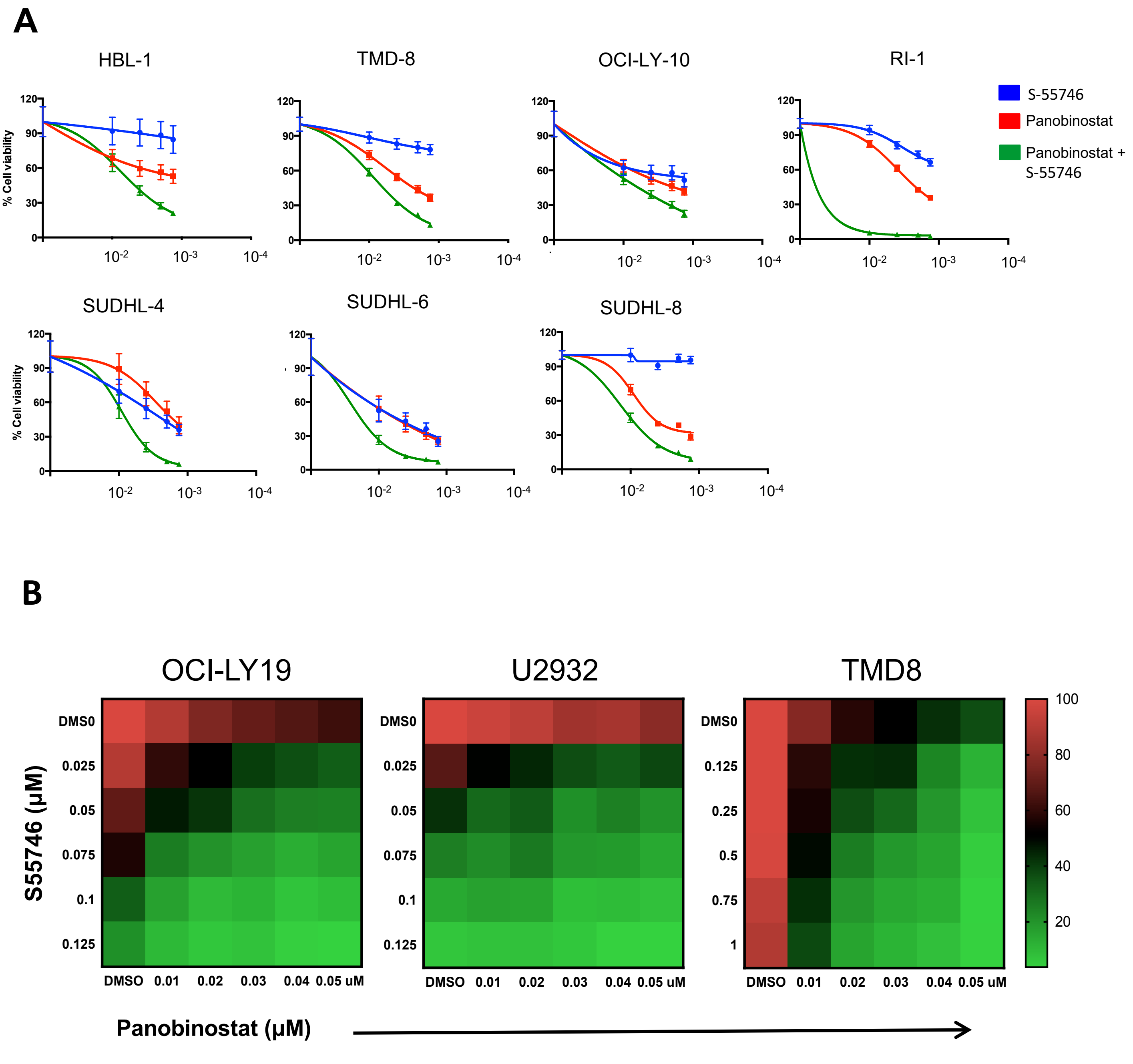

Figure S9. Panobinostat enhances the antiproliferative activity of the BCL2 inhibitor S-55746 in DLBCL cell lines.

A. MTS assay confirming enhanced antiproliferative effects of S-55746 in combination with panobinostat in DLBCL cells.

B. Heat maps showing synergistic effect of the combination of S-55746 and panobinostat in three representative DLBCL cells. Percentage of cell viability are depicted in a colorimetric scale from red (high) to green (low) normalize to DMSO (control). Values are the mean ± SD of three separate determinations. Cells were incubated with increasing concentrations of S-55746 and panobinostat for 24 hours and cell viability was determined by Celltiter-Glo assay.

Supplementary Figure 10

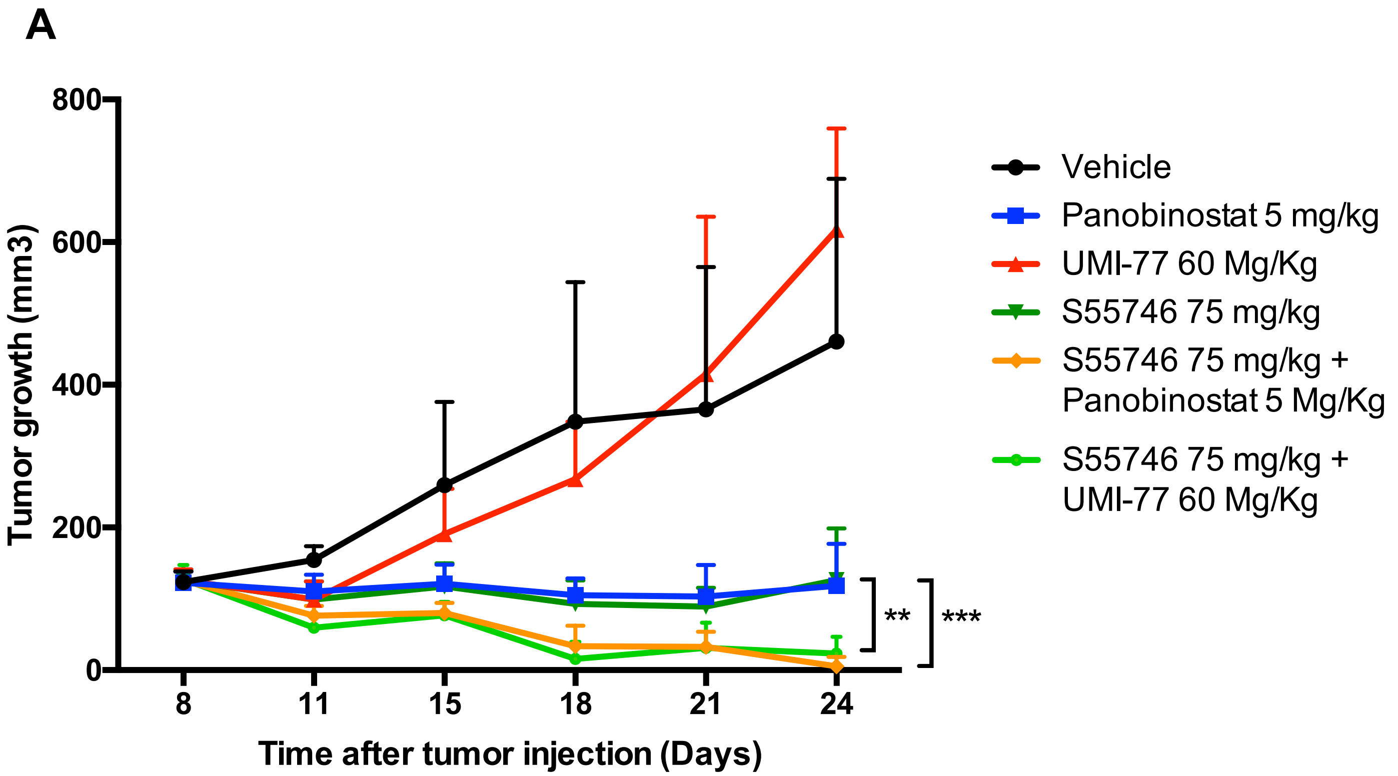

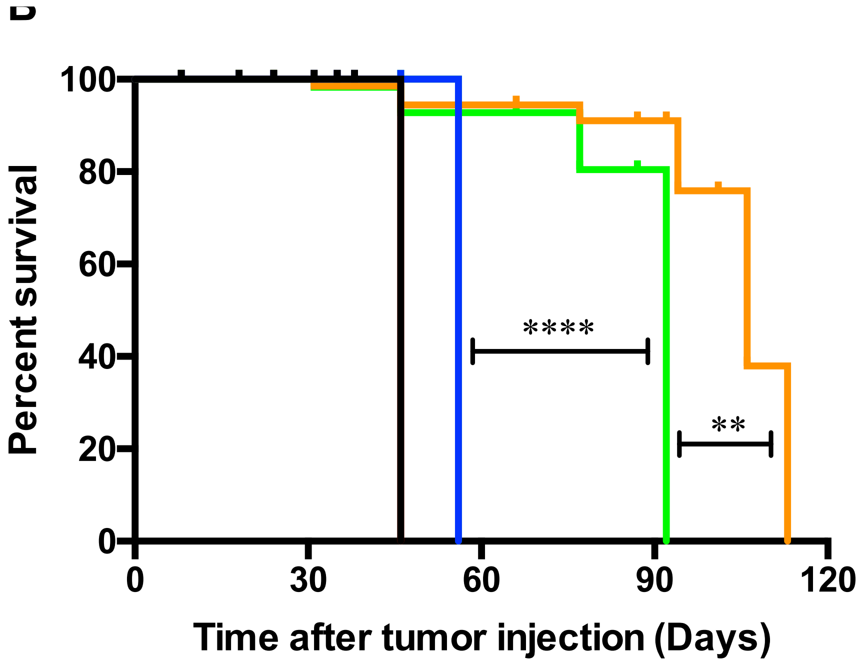

Figure S10. S-55746 synergizes with panobinostat in PDX DLBCL xenograft model.

A. NSG mice (n = 8 per treatment group) were injected with PDX DLBCL and treated with either vehicle, panobinostat (5 mg/kg 5 times weekly), UMI-77 (60 mg/kg every other day), S-55746 (75 mg/kg 5 times weekly) or the 2 drugs together for 3 weeks. Differences between groups were calculated with the ANOVA with Dunnett's test. $**P < 0.005$, $***P < 0.001$

B. Kaplan-Meier plot of the percent survival as a function of time from last drug administration. Data are from n = 8 for all groups in PDX DLBCL tumors. $**P < 0.005$, $****P < 0.0001$.

Supplementary Figure 11

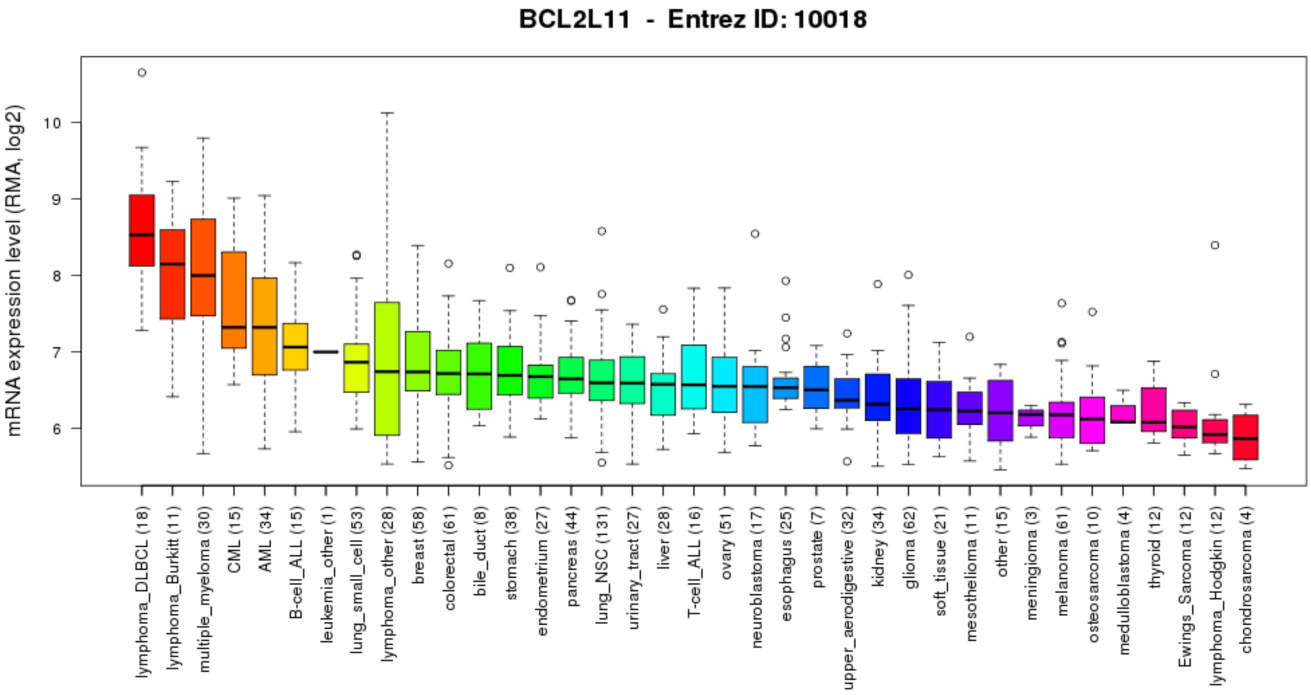

Figure S11. Publicly available BCL2L11 (encoding Bim) mRNA expression data archived in Cancer Cell Line Encyclopedia (http://www.broadinstitute.org/ccle/home).
